# Perceptions of risk in people with inflammatory arthritis during the COVID-19 pandemic

**DOI:** 10.1093/rap/rkac050

**Published:** 2022-06-20

**Authors:** Sarah Ryan, Paul Campbell, Zoe Paskins, Fay Manning, Katrina Rule, Michael Brooks, Samantha Hider, Andrew Hassell

**Affiliations:** Haywood Academic Rheumatology Centre, Midlands Partnership NHS Foundation Trust, Haywood Hospital, Stoke on Trent; School of Nursing and Midwifery, Keele University, Keele; Department of Research and Innovation, Midlands Partnership NHS Foundation Trust, St George’s Hospital, Stafford; School of Medicine, Keele University, Keele; Haywood Academic Rheumatology Centre, Midlands Partnership NHS Foundation Trust, Haywood Hospital, Stoke on Trent; School of Medicine, Keele University, Keele; School of Medicine, Keele University, Keele; Medical School, University of Exeter, Exeter, UK; Haywood Academic Rheumatology Centre, Midlands Partnership NHS Foundation Trust, Haywood Hospital, Stoke on Trent; Haywood Academic Rheumatology Centre, Midlands Partnership NHS Foundation Trust, Haywood Hospital, Stoke on Trent; Haywood Academic Rheumatology Centre, Midlands Partnership NHS Foundation Trust, Haywood Hospital, Stoke on Trent; School of Medicine, Keele University, Keele; Haywood Academic Rheumatology Centre, Midlands Partnership NHS Foundation Trust, Haywood Hospital, Stoke on Trent; School of Medicine, Keele University, Keele

**Keywords:** RA, COVID-19, health communications, perceptions of risk, qualitative research

## Abstract

**Objective:**

People with inflammatory arthritis have an increased incidence of serious illness and mortality, placing them at risk of poor outcomes from coronavirus disease 2019 (COVID-19). This study explored patients’ perceptions of risk from COVID-19 over a longitudinal period of the pandemic.

**Methods:**

Fifteen adults with inflammatory arthritis attending a National Health Service rheumatology service each took part in three semi-structured telephone interviews conducted between 16 September 2020 and 29 July 2021. Interpretive phenomenological analysis was undertaken by two researchers and two public contributors.

**Results:**

Four main themes relating to perceptions of risk from COVID-19 were identified: inflammatory arthritis; medications and co-morbidities; immediate social environment; health policy communication; and media influence. Participants recognized that having inflammatory arthritis increased their individual risk. Perceptions of risk and associated fear increased during the pandemic, influenced by family/friends who had had COVID-19 and health policy communications. The perceived constant use of negative messages led to many participants disengaging with the media. At the final interviews, when the vaccination programme was well established, participants continued to assess the risk and benefits of engaging in activities.

**Conclusion:**

This study demonstrates the breadth of factors that influenced perceptions of risk in people with an inflammatory arthritis. As health professionals, we have only a small sphere of influence over some of these factors, namely health-care communications. People with inflammatory arthritis appropriately knew that their condition increased their infection risk, but more could be done to consider how and to what extent we involve patients in explaining risk at times of crisis.

Key messages

- Patients were aware of their increased risk of coronavirus disease 2019 related to their condition and medications.
- Health-care communication on risk needs to contain clear, plain, neutral and active language.
- Involving patients in the content of health-care communication might improve understanding and reduce psychological distress.

## Introduction

Patients with inflammatory arthritis have an increased risk of serious illness, infection and death owing to their autoimmune condition, immunosuppressant medication and related co-morbidities [1]. At the onset of the coronavirus disease 2019 (COVID-19) pandemic, these factors were considered to make patients with inflammatory arthritis more susceptible to poorer outcomes from COVID-19 [2]. In a study of 17 million adults in the UK, the risk of COVID-19-associated death for a combined group of people with RA, SLE or psoriasis was slightly higher than that of the general population [3], with the risk of all-cause deaths more prominently raised in people with rare autoimmune diseases [4]. The risk of poor outcomes in people with RA from COVID-19 appears to be associated with co-morbidities, high disease activity and treatment with glucocorticoid CSs or rituximab [5].

On 21 March 2020, shielding was introduced in England for people considered to be particularly vulnerable to poor outcomes from COVID-19. Shielding involved being advised to stay at home and avoid all face-to-face contact outside their household for 12 weeks [6]. In response to government policy, the British Society for Rheumatology formulated risk-stratification criteria to identify patients who needed to shield, based on a combination of age, medication and co-morbidities [2]. Rheumatology departments were asked to help identify and contact those who were identified as being at increased risk of poor outcome from COVID-19 to reinforce government messaging.

People with inflammatory arthritis have had to evaluate and assess their own risk over the various stages of the pandemic (including lockdown periods), the changes to government policy and the introduction of the vaccination programme. This longitudinal study explored perceptions of risk during the COVID-19 pandemic in people with inflammatory arthritis.

## Methods

The theoretical framework for the study was interpretative phenomenology. The aim of interpretative phenomenology is to understand the experience of the person and to uncover the meaning of the experience for the individual. This approach enables the participants, in this case people with inflammatory arthritis, to describe their perceptions and experiences of their individual level of risk during the COVID-19 pandemic [7]. The reporting of this study was based on The Consolidated Criteria for Reporting Qualitative Health Research [8].

### Participant selection

Patients with inflammatory arthritis, predominantly RA, were recruited from a rheumatology department in a community hospital in Staffordshire. Eligible patients were identified from a rheumatology clinical database and were sampled purposively to ensure a representation of age, biological sex, shielding and non-shielding status. To obtain a sample size of 15–20 patients, 40 patients were mailed an expression of interest letter inviting them to participate. If a positive response was received, a consent form and participant information sheet were posted. Fifteen patients returned an expression of interest form and participated in the study. There is no definitive sample size for a qualitative study, but to embrace its ideographic commitment, smaller concentrated samples are commonly used [9]. The rationale for the sample size was influenced by the longitudinal study design [10].

All patients with RA were sent a letter from the rheumatology service. This contained information regarding the need to continue with current medications; a scoring grid to assess levels of individual risk (based on the BSR risk stratification criteria [2]) with actions to take if an individual was at high risk; measures to take if COVID-19 symptoms occurred; reinforcement of the government’s public health advice; guidance on maintaining emotional wellbeing; and Web links to patient organizations.

### Ethical approval

Ethical approval was granted by Camden and Kings Cross Research Ethics Committee REC reference: 20/HRA/3406. Written consent (email consent for those participants who were shielding and could not use the postal service) was obtained and reconfirmed before the interviews.

### Data collection

**Fig. 1:**
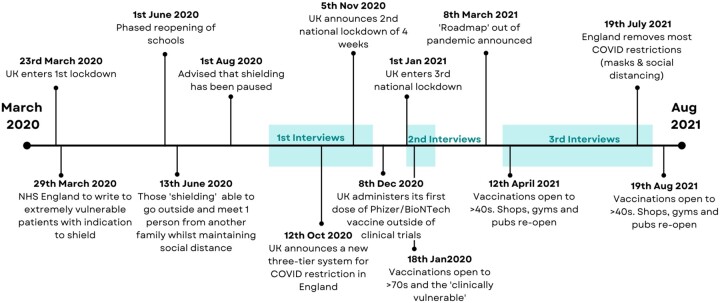
Time line of events in the UK pandemic response in relationship to the study interviews The tier system involved a series of regional public health restrictions based on the incidence of coronavirus disease 2019 (COVID-19) within the locality.

Participants engaged in three semi-structured telephone interviews with the same interviewer (P.C.), who was not known to participants before the first interview. Interviews were conducted at baseline (16 September–23 November 2020), at 2–4 months (11–27 January 2021) and finally at 6–10 months (27 April–29 July 2021). Fig. 1 indicates when the interviews were performed and the prevailing restrictions at the time.

The initial topic guide was developed to examine patients’ experiences of living with inflammatory arthritis during the pandemic and reviewed by members of the study group (S.R., P.C., A.H., Z.P., S.H. and F.M.) and two patient partners (K.R. and M.B.), then refined further after two pilot interviews. Given that patients’ perceptions of risk were identified as key areas, these themes were developed iteratively. Subsequently, the second and third interviews used the participants’ previous narrative to explore perceptions of risk in relationship to significant events, including lockdowns, shielding and the vaccination programme (see Fig. 1) during the pandemic. The topic guides are available in Supplementary Data S1–S3, available at *Rheumatology Advances in Practice* online.

Demographic data, including biological sex, age, disease duration, occupational and marital status, were collected. The interviews were digitally recorded, transcribed verbatim and anonymized.

### Data analysis

Interpretative phenomenological analysis was undertaken by two members of the research team (S.R. and P.C.). There is no single definitive approach to data analysis in interpretative phenomenological analysis, and to assist with validity and rigour we used an inductive method with coding and theme development directed by the content of the data, as advocated by Braun and Clarke [11]. Each transcript was read repeatedly to ensure familiarization with the data and to generate initial codes to identify specific patterns of meaning. Over the course of three research group meetings, themes were identified from the coded data. (See Supplementary Data S4, available at *Rheumatology Advances in Practice* online, for data analysis.) The findings were shared with the two patient partners to inform the interpretation of the data further.

### Patient and public involvement

Two people with lived experience of inflammatory arthritis (K.R. and M.B.) were involved in all stages of the research. This included the design of the study (interviews rather than focus groups), reviewing the public-facing information (invitation letter, patient information sheet and consent form), informing the content and piloting of the topic guides and data analysis.

## Results

Fifteen patients participated in the three planned interviews (resulting in a total of 45 interviews). Fourteen had a diagnosis of RA and one had a diagnosis of adult-onset Still’s disease. Disease duration was an average of 22 ± 13 years (range 1.5–46 years). The sample included nine females and six males, with ages ranging from 46 to 79 years. The majority of participants were retired (*n* = 10), with one currently out of work and the remaining employed (*n* = 4). All participants were Caucasian, and 11 of the 15 participants were married (see Table 1). The interviews lasted between 23 and 60 min. Data saturation was achieved, with no new concepts arising after the 10th participant was interviewed. Four main themes were identified, which related to perceptions of risk in people with RA during the pandemic: inflammatory arthritis, medications and co-morbidities; immediate social environment; health policy communications; and media influence. The themes are discussed below, with supporting illustrative quotes relating to the three interview time points, shown in Table 2.

**Table 1:** Characteristics of participants

Characteristic	Number of participants
Sex	
Female	9
Male	6
Diagnosis	
RA	14
Adult Still’s disease	1
Age, years	
40–49	2
50–59	3
60–69	3
70–79	7
Medication, number of medications prescribed (some participants were taking a combination of therapies)	
Conventional DMARDs	15
Biologic DMARDs	12
Glucocorticoid	4
Shielding status	6
Non-shielding status	9
Disease duration, years	
1–10	4
11–20	2
21–30	5
>30	4
Occupational status	
Retired	10
Working	4
Currently not working	1
Marital status	
Married	11
Living with partner	3
Single	1

**Table 2:** Results

Theme 1: having inflammatory artthritis		
**Interview 1, September**–**November 2020**	Interview 2, January 2021	**Interview 3, April**–**July 2021**
‘I do know I have to be careful with my immune system I’ve always known that because it’s an immune condition RA is.’ (Female aged 61 years)	‘I know I’ve had the biologics and it’s reduced my immune system, so I do I feel as though, because of the meds and everything that I’m on, I’ve got not resistance. If something was to come my way, I wouldn’t be able to fight it.’ (Female aged 57 years)	‘I mean, they ask me to go out bowling on Thursday night, well one I’d just had my infusion [rituxamib] on the Wednesday, so by rights I should be being a bit careful and shielding.’ (Male aged 55 years)
‘I never actually thought I could be at more risk because of the steroids.’ (Male aged 55 years)	‘It means that if we contact COVID in any sort of way within a week we would probably be in hospital in intensive care and we would die because of the drugs we’re on.’ (Female aged 73 years)	‘Well, I had to shield again because of the steroid injection, so I won’t be able to go out.’ (Female aged 57 years)
‘A certain resentment, really, at the condition that I’d got could make me more prone to not only catching the virus but having potentially a worse result after catching the virus. I felt a little bit of resentment that despite my best efforts something might get me that was totally out of my control.’ (Male aged 55 years)	‘It’s made me realize that it’s the medication, because its immunosuppressant, it’s made me realize that the medication I’m on is a serious one.’ (Female aged 78 years)	‘I’ve been put on steroids, but I think they’re very reluctant to put people on steroids because of the risk of infection, so it heightens your risk, but I could be completely wrong about that.’ (Female 61 years).
‘I shouldn’t be frightened, by something as small as COVID I recognize is going to kill me, so I am nervous. I don’t want to die yet.’ (Male aged 71 years)	‘So, I do very much consider myself to be extremely vulnerable. Now that’s a combination of having been ill and medication and RA all rolled in together.’ (Male aged 71 years)	‘Yes. I know people say, oh you get the vaccination, but you never know if it’s actually going to work with you, do you, especially me having the arthritis stuff, they were never sure whether it would work or not anyway.’ (Male aged 66 years)
‘As soon as I heard that people develop breathing difficulties, I was only a few months off the pneumonia, knew that if I caught this COVID I would be dead. I would not survive it.’ (Male aged 71 years)	‘I saw the haematologist, and she said, whatever you do don’t get ill, and so that’s always praying on my mind. I think if I get ill it’s going to be bad.’ (Male aged 71 years)	‘You’ve had the vaccine but you can still catch it, and nobody knows for people like me who are immunosuppressive or have got any other sort of problem, they don’t know if you catch it, is it still going to be bad or not.’ (Female aged 71 years)
‘So, I knew I had to be sort of extra careful because I’ve got a bit of asthma as well.’ (Female aged 71 years)		

Theme 2: immediate social environment		
**Interview 1, September**–**November 2020**	Interview 2, January 2021	**Interview 3, April**–**July 2021**
	‘We know people or of people who’ve had it, and that’s led us to think that this is getting more serious, you know, this latest one is worse.’(Female aged 75 years)	‘No, I am very wary. My cousin got COVID and ended up in hospital, and her husband who was very, very fit, he got it and he almost died. It was a serious wake-up call, so yes, we have been ultra-careful because of that, and we’re probably going to be the last couple in England who will be out and about.’ (Female aged 75 years)
	‘I think I’m a lot more frightened of the virus than I was. I think obviously the numbers going up, I think because I personally know a lot more people, I think it was a bit more remote certainly during the first lockdown.’ (Female aged 61 years)	‘I think I still would prefer not to mix with lots of people at the club, because the ones who are going back are younger people who probably haven’t been terribly careful because they don’t feel threatened.’ (Female aged 67 years)
	‘A neighbour had got it. He’s on the ventilator now, so it does bring it home, doesn’t it? Whereas before it was just something in the paper, and now its people that you know or relatives, or whatever.’ (Male aged 66 years)	‘Normally, we go away each year with friends, and sometimes we travel together to France, you see, in the same vehicle, but I can’t imagine wanting to do that.’ (Female aged 57 years)
	‘I think (the fear has reduced) as well because my daughter’s friend had it and he’s OK.’ (Male aged 54 years)	‘I used to use the buses a lot, but I haven’t been on a bus, and I don’t think I’d be on one yet.’ (Female aged 73 years)
	‘A very close friend just walked straight into the house. I think she realized that we were uncomfortable. I’m not a great risk taker really.’ (Female aged 61 years)	‘Well, I am still extraordinarily careful. I’ve been to some garden centres.’ (Male aged 71 years)
	‘The risk has changed because of the new variants that are 70% more transmittable. It’s put me on the back foot more so than it did before.’ (Male aged 72 years)	‘I just got to the point of thinking, well, if I trust the people I’m going to see and if they’ve had negative tests before I see them then I want to exercise my choice.’ (Female aged 78 years)
	‘I think it will probably be gently, gently with us. When more people have been vaccinated, maybe we might go out with a couple of friends and have a meal. Certainly not interested in going on a plane yet or a cruise definitely, definitely not yet, not for quite a while.’ (Female aged 75 years)	‘We’ve been to the dentist and opticians, but as for socializing, no we haven’t started doing anything like that yet. We don’t want to go out for meals yet; we don’t feel that the time is right yet.’ (Female aged 79 years)
		‘It’s a question of striking a balance between mental wellbeing and protecting myself physically, and I just thought, well, I’m doing the best I can. I don’t think I’m putting myself at risk, and I need to go out and do things.’ (Female aged 67 years)

Theme 3: formal health policy communication		
**Interview 1, September**–**November 2020**	Interview 2, January 2021	**Interview 3, April**–**July 2022**
‘Then had a letter from the hospital and it explained in details the significant risks that were present and how certain drugs just increase the risks and combination of drugs pushed it even further, and so we recognized that we were both in a very high risk category.’ (Male aged 72 years)	‘The risk has changed. Because I’m clinically extremely vulnerable, it’s put me on the back foot, more so than it did before.’ (Male aged 72 years)	‘That affected me [being classified as clinically extremely vulnerable] and made me very risk averse.’ (Female aged 67 years)
‘I got a letter from the NHS saying I’m very vulnerable, so I then took it far more seriously and then I got bombarded with texts and letters from the NHS and my GP and it frightened the life out of me.’ (Female aged 75 years)	‘I don’t think clinically extremely vulnerable is sufficient for the layman generally speaking. It needs to have short, sharp, punchy words like “high risk, very high risk”, you know, so I feel that people would be a bit more understanding.’ (Male aged 72 years)	‘I think in some cases it’s almost I get to a point where I’m feeling overwhelmed with being shut off and isolated, so then I sit down and talk to myself and say, right, what’s the risk and how far are you at risk if you do this and how much will it matter if you don’t do it? And then I just say, right, this is what I’m going to do, this is what I’m happy with doing.’ (Female aged 67 years)
‘I had a letter from The Haywood, and it did state that I was vulnerable and should be careful, and it gave a check list of things to tick off, and then it said, if you have one tick you’re low, if you have two tick’s you’re medium, if you have three ticks you’re high vulnerability, and I kind of thought, well, yes, OK, I understand that, but I’m not going to stay in and avoid life for the next 6 months or the next year.’ (Female aged 67 years)	‘Now you’re thinking, yes, it’s a nice day, but if I go here what risk am I taking? So your brain’s working overtime all the time, thinking of what ifs and is it safe?’ (Male aged 66 years)	‘Doesn’t feel any safer with having both vaccines. I think it’s only going to take time. I think it’s just got to be a natural progression of numbers being down, feeling a little bit more relaxed with everything. I think it’s just time.’ (Female aged 57 years)
‘I knew it was too risky to go out, so I didn’t. I stopped doing absolutely everything.’ (Female aged 61 years).	‘The vaccine has delivered a layer of confidence. Without that, I would not be leaving the house.’ (Male aged 71 years)	‘After the vaccine, I was much more confident to take a risk and go out, using all the measures what is it, “hands, face, space”, all of that doing and those things but do you know, I wouldn’t have even done them without the vaccines.’ (Male aged 71 years)
‘I did a kind of modified shielding in that I didn’t completely isolate. I was still going out and about, but I wasn’t going to crowded places, I stopped doing the shopping, I was very selective about where I went, and that, I think, was a protective factor in terms of physical and psychological health.’ (Male aged 47 years)	‘I’ve had four letters now from Matt Hancock telling me I’m clinically extremely vulnerable, and I didn’t really like it but I’ve decided to use it to my advantage, if it means that I will get vaccinated earlier because of having that label.’ (Female aged 67 years)	‘I think we’ve become very risk averse, haven’t we? And we’ve kind of changed as a nation really. People are so frightened, you know. I think everybody takes risks at some point, don’t they?’ (Female, aged 61 years)

Theme 4: media influence		
**Interview 1, September**–**November 2020**	Interview 2, January 2021	**Interview 3, April**–**July 2021**
**‘**On the news and hearing on the television about large numbers of people dying, it really did bring it home that something very profound was happening.’ (Male aged 71 years)	‘Well, it’s all been negative, negative, negative all of the time. There should have been far more positive messages that if you do this, this will be the outcome.’ (Male aged 72 years).	‘Well, I stopped watching the news. I read the newspaper, and that’s about it. I don’t watch any news anymore because I found it was just too depressing.’ (Male aged 66 years)
‘But as soon as the world was aware of it, news bulletins were gearing everybody to expect a tsunami of illness crashing onto the shore.’ (Male aged 72 years)	‘It hasn’t changed my behaviour. I basically don’t look at the news any more on the television ’cos I feel that that’s just basically negative all the time.’ (Male aged 65 years)	‘Everything seems to be doom and gloom even though we’re getting more and more people vaccinated and there’s still doom and gloom on the telly.’ (Male aged 65 years)
‘The papers were very doomsday kind of thing, weren’t they? They’d go from one extreme to the other.’ (Male aged 65 years)	‘I try not to watch it, because one day they’ll say one thing and then the next day they say something else.’ (Male aged 66 years)	‘I have to keep checking when can I do this and you know ’cos it’s different for every country as well, isn’t it? Like Scotland and Wales are slightly different. So, I would say stop telling us the rules in Scotland and Wales because then I get really confused.’ (Female aged 61 years)
‘Watching on the news, seeing television programmes, hearing on the radio the numbers of people dying from COVID, especially when you have a weakness, is horrifying, and I would not venture out.’ (Male aged 71 years)	‘I actually think the media are just as responsible as the people who don’t adhere to the guidelines, because they’re so negative, the media has made it confusing for people.’ (Female aged 71 years)	‘I don’t think even my husband is watching the news so much now.’ (Female aged 67 years)
	‘The way the media has portrayed the vaccine has been good because they have highlighted people getting it and talked about the Queen getting it, and it does influence people, you know, seeing all these people lining up to get the vaccine.’ (Female aged 61 years)	‘It’s all about whether people can fly to Spain on holiday, and it does feel like the death and destruction headlines have been replaced with back to normal, just griping at everyone, which feels better.’ (Female aged 46 years)

### Theme 1: inflammatory arthritis, medications and co-morbidities

During the first two interviews, all participants referred to their increased risk of contracting COVID-19 and the likelihood of serious consequences if they contracted the virus, which they attributed to having RA and taking DMARDs and/or biologic therapy. For some participants, the risk associated with their medications reinforced the ‘serious nature’ of their drug therapy. Most participants were, initially, less aware of the risks associated with taking CSs.

The majority of participants recognized that having other medical conditions, such as asthma or cancer, increased their risk of serious outcomes if they contracted COVID-19. Concern was also expressed regarding whether the vaccination would be effective with having inflammatory arthritis. Having inflammatory arthritis led to some participants expressing concerns about their mortality. One participant expressed resentment that having RA reduced their ability to influence the level of risk they faced. By the final interview, most participants were assessing the risks of engaging in social activities after receiving drug treatments, including rituximab.

### Theme 2: immediate social environment

The importance of the immediate social environment became more evident during the second interviews. As case numbers became higher, new variants were discovered, and many participants personally knew of family and friends who had had COVID-19. All these factors led to increased perceptions of risk. However, for one participant, knowing someone who had recovered from COVID-19 helped to reduce their fear.

In the second and third interviews, some participants were uncomfortable when friends were deemed as taking an unnecessary risk by entering a house uninvited. Another participant based her own assessment of risk on the trust she had in the people she met, based on the understanding that they had performed a lateral flow test.

In the final interviews, the significance of the virus continued to be felt when family members who were younger and fitter became seriously ill with COVID-19, re-emphasizing that risk was still present. Although the vaccination programme was active by the third interviews and lockdown had ended, most participants were still hesitant in recommencing social activities, especially if it involved younger people, because it was perceived that younger people might not have taken the pandemic seriously. Some activities, such as travelling abroad or using public transport, were considered too high risk to engage in, whereas visiting a garden centre or attending for health care (dentist and optician) had less risk attached to them. Some participants balanced the perceived risk of going out with the need to maintain mental wellbeing.

### Theme 3: health policy communication

During the first two interviews, all participants’ level of fear and the risk of contracting COVID-19 increased following correspondence from NHS England, their rheumatology department and general practitioners. This fear led to many participants conforming to shielding requirements, whereas other participants, despite being mindful of their need for safety, introduced a modified form of shielding to protect their physical and psychological wellbeing. Some participants found it stressful constantly to assess the level of risk attached to a particular behaviour.

During the second interviews, with England entering its third lockdown, the impact of being categorized as clinically extremely vulnerable increased perceptions of risk and influenced participants to adopt risk-averse behaviours. There was concern that the term clinically extremely vulnerable was complex and difficult to understand.

The development of the vaccination programme offered participants hope that by increasing immunity their risk of becoming seriously ill would be reduced. During the second interviews (when some participants had received their first vaccination), most participants reported initially feeling safer and more confident in their behaviours. At the third interview, despite all participants being vaccinated, caution was still exercised around behaviour, and this would continue until more restrictions were lifted and a greater number of the population had been vaccinated.

### Theme 4: media influence

During the first interviews, media reports and the UK government evening briefings, including the number of daily deaths, made the risk of COVID-19 very apparent and influenced the behaviour of many participants to stay at home. During the second interviews, perceptions of constant negative media messages contributed to many participants feeling low and alienated from the media. All participants expressed a desire for clearer communications and a greater focus on positive events, citing the success of the vaccination programme. Focusing more on reports that showed role models being vaccinated was regarded as one way the media could influence the further uptake of vaccines. By the final interviews, the focus on ‘death and destruction’ in the media was slowly being supplemented with information about air travel, providing some semblance of normality to some participants.

## Discussion

This study explored the perceptions of risk in people with inflammatory arthritis during the coronavirus pandemic, using longitudinal interviews. Perceptions of risk remained high throughout all interviews, which might reflect the scarcity of evidence regarding the precise estimate of risk in this population during the duration of the study. Earlier findings from this study demonstrated that the main impact of the pandemic on wellbeing related to emotional status [12].

Key findings were the awareness of risk that participants had at the start of the pandemic attributable to having inflammatory arthritis and receiving drug treatment for the condition. Perceptions of risk increased as the pandemic progressed, influenced by friends and family who had had COVID-19 and reports of new variants of the virus. Health policy communications and media reporting heightened perceptions of risk. At the final interviews, when the vaccination programme was well established and most COVID-19 restrictions in England had been lifted, participants still adopted a cautious approach and continued to assess the risk and benefits of engaging in social activities.

A key strength of this study is the longitudinal design. Participants were interviewed at three time points over a period of 6 months, during which there were significant changes in COVID-19 prevalence, mortality and health policy in the UK. This has enabled us to gain insight into patients’ perception of risk over time, giving us greater perspective. The involvement of research group members and two patient partners in coding and interpreting the data enhances the credibility of the findings. There are several limitations of the study. Firstly, given that the initial interviews occurred after the first lockdown in the UK, the participants’ reflections at this point occurred retrospectively; and secondly, the majority of the sample was not in employment, and consequently, the impact of work could not be explored fully. Although the participant sample in this study included a range of age at disease onset (20–66 years old), disease duration (1.5–46 years) and of biological sex, the sample was primarily of older individuals and solely of Caucasian ethnicity, which broadly reflects the population of people with inflammatory arthritis in North Staffordshire. Further research focusing on a more diverse sample of patients would be beneficial in understanding the wider perceptions of risk throughout the pandemic.

At the start of the pandemic, our participants recognized their increased risk from COVID-19, attributed to having inflammatory arthritis, medication use and co-morbidities. All participants were aware that RA was an autoimmune condition and that medications could compromise their resistance to infection. Similar attributions were demonstrated in a survey of 550 people with a rheumatic disease, who rated medications as their top concern (76.1%) in increasing the severity of COVID-19 [13], whereas patients with lupus had high levels of anxiety regarding their mortality risk to COVID-19 [14]. It is not known how our participants obtained their knowledge of the risk associated with having inflammatory arthritis and medication use. Patients commencing DMARDs commonly attend for an information session with a rheumatology nurse or pharmacist to learn about the benefits and side effects of DMARD treatment. The information gained from these sessions might have resonated with patients at the start of the pandemic. Some participants initially expressed surprise that CSs could also increase their infection risk.

The fear of having COVID-19 increased during the second interviews owing to the discovery of new variants, higher prevalence of COVID-19 in the community, and knowing friends and family who had had COVID-19. A cross-sectional study of older adults in Bangladesh that aimed to assess the perceived fear of COVID-19 and its associated factors showed that having a close friend or family member diagnosed with COVID-19 was associated with a significant rise in fear [15].

Receiving communication from official sources, including NHS England, the rheumatology department and general practitioners, clarifying risk status increased fear and heightened individual perceptions of risk in the majority of participants. Health communications that start by fostering wellbeing have the potential to promote effective and sustainable behavioural change during the pandemic and might help to reduce potential fragmentation of risk behaviour [16]. Presenting the risk attributed to having RA without offering clear options for how to lower the risk might induce psychological distress and affect patients’ understanding of their potential risk [17, 18]. Our participants found receiving written information from different official sources (rheumatology department and the government) overwhelming. This could have related to the depth of the written communication (the letter from the rheumatology department was seven pages), and public health messages regarding how risk could be reduced might not have been easy to interpret alongside all the other information. Therefore, it might be helpful to commence communications relating to a health risk with the actions that patients can take to address the risk, whilst ensuring that the actions recommended are clear and easy to locate.

Certainly, psychological distress was a factor entwined with participants’ assessment of risk and associated fear. However, variation was present in how this was managed between participants and across time points. One particularly relevant theory can be applied to understand this variation. Terror management theory [19] encapsulates cognitive processes when individuals face extreme threat of death (terror). The theory argues that the resulting anxiety is cognitively managed or buffered through shared beliefs and standards about reality, self-esteem from those beliefs, all validated by the person’s close personal relationships. Terror management theory has been applied to understand the heterogeneity of reactions to COVID-19 [19] and how people balance the threat of death with the need to maintain a life that has value and meaning. This negotiation of risk *vs* meaningfulness might be reflected in our findings of some participants who introduced a modified form of shielding to preserve some form of social interaction, in an attempt to maintain their wellbeing. Such behaviours have been shown in other similar illness groups; for example, where people with lupus left the house and sought social interaction to reduce the impact on their mental health [14].

The fear of having COVID-19 might have influenced positive vaccine behaviour because, by the time of the final interviews, all our participants had been vaccinated. This reflects the results of a Finnish survey, which found that those perceiving COVID-19 as a severe disease were more likely to have the vaccine [20]. Although the vaccination programme provided hope and increased confidence, after vaccination the participants still assessed the risk of engaging in specific activities.

Over the course of the pandemic, participants disengaged with the media. This reduction in media consumption was also reflected in the general public [21]. Su *et al.* [22] proposed that an effective media crisis communication strategy should be fact based and people centred, including the delivery of facts that matter to the people without framing the numbers/statistics based on personal views or ulterior motives. This is important, because effective risk communication is crucial for understanding health threats and supporting people in making informed decisions for mitigating the risks [23, 24].

There are several implications for practice arising from this study. Firstly, there is a need for patient input into the content of communications regarding risk even in a crisis, to ensure that risk is communicated in such a way as to promote behavioural changes without inducing unnecessary fear or causing psychological distress. This can be addressed by providing information that uses clear, plain, neutral and active language; for example, ‘if you take this action’. [25] Gigerenzer *et al.* [26] described the concept of ‘collective statistical illiteracy’, referring to a large proportion of the population who lack the ability to understand and interpret numbers, often used during the pandemic to convey risk. Using pictographs might be one way of helping patients to make unbiased decisions regarding their individual risk [25].

Receiving communications from a number of different official sources, including the rheumatology service and the government, increased the fear experienced and was clearly overwhelming for some participants. We do not know whether patients were able to complete their assessment of risk accurately using the scoring system they were sent. If an overestimation of risk occurred in some instances, this could have increased feelings of distress. Commencing written communication with positive messages on actions that patients can take might assist in mitigating some of the risk. Further research is required to explore how we can communicate health messages to patients in an effective, balanced manner without causing heightened feelings of distress.

Secondly, information regarding CS use might not be given in the same standardized format as education on DMARDs, and the need for more detailed information regarding the benefits and limitations of CSs is something that health professionals should consider. Thirdly, telephone support from health professionals or ongoing peer support from trained volunteers could be used to address fears and support patients [27] with inflammatory arthritis in their decision-making regarding the potential risk of engaging in certain behaviours, to provide some sense of control.

### Conclusion

This study demonstrates the breadth of factors that influenced perceptions of risk in people with inflammatory arthritis. As health professionals, we have only a small sphere of influence over some of these factors, namely health-care communications. People with inflammatory arthritis appropriately knew that their condition increased their infection risk, but more could be done to consider how and to what extent we involve patients in explaining risk at times of crisis.

## Supplementary Material

rkac050_Supplementary_Data

## References

[1] Luqmani R, Hennell S , Estrach C. BSR/BHPR rheumatology guidelines for the management of rheumatoid arthritis. Rheumatology 2006;56:865–8.

[2] Price E , MacPhie E, Kay L et al Identifying rheumatic disease patients at high risk and requiring shielding during the COVID-19 pandemic. Clin Med 2020;20:256–61. 10.7861/clinmed.2020-0149 PMC7354033 32371418

[3] Williamson EJ , Walker AJ, Bhaskaran K et al Factors associated with COVID-19-related death using OpenSAFELY. Nature 2020;584:430–6. 32640463 10.1038/s41586-020-2521-4 PMC7611074

[4] Peach E , Rutter M, Lanyon P et al Risk of death among people with rare autoimmune diseases compared with the general population in England during the 2020 COVID-19 pandemic. Rheumatology 2021;60:1902–7. 33271595 10.1093/rheumatology/keaa855 PMC7798585

[5] Grainger R , Kim AHJ, Conway R, Yazdany J, Robinson PC. COVID-19 in people with rheumatic diseases: risks, outcomes and treatment considerations. Nat Rev Rheumatology 2022;18:191–204. 35217850 10.1038/s41584-022-00755-x PMC8874732

[6] Department of Health & Social Care. Guidance on shielding and protecting people who are clinically extremely vulnerable from COVID-19. 2020. https://www.gov.uk/government/publications/guidance-on-shielding-and-protecting-extremely-vulnerable-persons-from-covid-19 (7 October 2021, date last accessed).

[7] Smith JA , Osborn M. Interpretative phenomenological analysis as a useful methodology for research on the lived experience of pain. Br J Pain 2015;9:41–2. 26516556 10.1177/2049463714541642 PMC4616994

[8] Tong A , Sainsbury P, Craig J. Consolidated criteria for reporting qualitative research (COREQ): a 32-item checklist for interviews and focus groups. Int J Qual Health Care 2007;19:349–57. 17872937 10.1093/intqhc/mzm042

[9] Smith JA , Shinebourne P. Interpretative phenomenological analysis. Washington DC: American Psychological Association, 2012.

[10] Morse JM. Determining sample size. Qual Health Res 2000;10:3–5.

[11] Braun V , Clarke V. Using thematic analysis in psychology. Qual Res Psychol 2006;3:77–101.

[12] Ryan S , Campbell P, Paskins Z. Exploring the physical, psychological and social wellbeing of people with rheumatoid arthritis during the coronavirus pandemic: a single centre, longitudinal, qualitative interview study in the UK. BMJ Open 2021; In press. 10.1136/bmjopen-2021-056555 PMC9330330 35882463

[13] Antony A , Connelly K, De Silva T et al Perspectives of patients with rheumatic diseases in the early phase of COVID‐19. Arthritis Care Res 2020;72: 1189–95. 10.1002/acr.24347 PMC7300883 32526068

[14] Sloan M , Gordon C, Lever E et al COVID-19 and shielding: experiences of UK patients with lupus and related diseases. Rheumatol Adv Pract 2021;5:rkab003. 33728396 10.1093/rap/rkab003 PMC7928599

[15] Mistry SK , Ali ARMM, Akther F et al Exploring fear of COVID-19 and its correlates among older adults in Bangladesh. Global Health 2021;17:1–9. 33853616 10.1186/s12992-021-00698-0 PMC8045579

[16] Porat T , Nyrup R, Calvo RA et al Public health and risk communication during COVID-19—enhancing psychological needs to promote sustainable behavior change. Front Public Health 2020;8:637. 10.3389/fpubh.2020.573397 PMC7652763 33194973

[17] Stack RJ , Stoffer M, Englbrecht M et al Perceptions of risk and predictive testing held by the first-degree relatives of patients with rheumatoid arthritis in England, Austria and Germany: a qualitative study. BMJ Open 2016;6:e010555. 10.1136/bmjopen-2015-010555 PMC4932277 27357193

[18] Fischhoff B. Communicating risks and benefits: an evidence based user's guide. US Department of Health: Government Printing Office, 2012.

[19] Pyszczynski T , Lockett M, Greenberg J et al Terror management theory and the COVID-19 pandemic. J Human Psychol 2021;61:173–89. 10.1177/0022167820959488 PMC7498956 38603072

[20] Karlsson LC , Soveri A, Lewandowsky S et al Fearing the disease or the vaccine: the case of COVID-19. Pers Individ Dif 2021;172:110590. 33518869 10.1016/j.paid.2020.110590 PMC7832025

[21] Nielsen RK , Kalogeropoulos A, Fletcher R. Most in the UK say news media have helped them respond to COVID-19, but a third say news coverage has made the crisis worse. Oxford: Reuters Institute for the Study of Journalism, 2020: 25.

[22] Su Z , McDonnell D, Wen J et al Mental health consequences of COVID-19 media coverage: the need for effective crisis communication practices. Global Health 2021;17:4–8. 33402169 10.1186/s12992-020-00654-4 PMC7784222

[23] World Health Organization. Risk communication and community engagement readiness and response to coronavirus disease (COVID-19): interim guidance, 19 March 2020. World Health Organization, 2020:WHO/2019-nCoV/RCCE/2020.2. https://apps.who.int/iris/handle/10665/331513 (30 June 2022, date last accessed)

[24] Lowbridge CP , Leask J. Risk communication in public health. New South Wales Public Health Bull 2011;22:34. 10.1071/NB10055 21527080

[25] Stacey D , Légaré F, Lewis K et al; Cochrane Consumers and Communication Group. Decision aids for people facing health treatment or screening decisions. Cochrane Database Syst Rev 2017;2017:https://doi.org/10.1002/14651858.CD001431.pub5. 10.1002/14651858.CD001431.pub5 PMC6478132 28402085

[26] Gigerenzer G , Gaissmaier W, Kurz-Milcke E et al Helping doctors and patients make sense of health statistics. Psychol Sci Public Interest 2007;8: 53–96. 26161749 10.1111/j.1539-6053.2008.00033.x

[27] O’Connell ME , Haase KR, Grewal KS et al Overcoming barriers for older adults to maintain virtual community and social connections during the COVID-19 pandemic. Clin Gerontol 2022;45:159–13. 34233600 10.1080/07317115.2021.1943589

