# Supplementary material for: Perceptions of risk in people with inflammatory arthritis during the COVID-19 pandemic
Source: Rheumatol Adv Pract. 2022 Jun 20;6(2):rkac050. doi: 10.1093/rap/rkac050 (PMC9255274; doi:10.1093/rap/rkac050)
Supplement: rkac050_Supplementary_Data [file rkac050_supplementary_data.zip › 22-028 Supplementary Data S2 - 2nd stage interview guide.docx]

**Topic Guide**

**The experience of living with Inflammatory Arthritis during the Coronavirus Pandemic**

**Preamble**

- Since our last interview on (date of previous interview):
  - How, if any, has your arthritis changed since our last interview (consider increase in symptoms, impact on physical, psychological and social function)?
- Has the way you have been managing your arthritis changed since we last spoke
- Some people we have talked to are working (e.g. in employment, or dong voluntary work), if that applies to you, can you tell me your experiences since the last interview?
- How did you feel during the period of national restrictions in November that were similar to the main lockdown earlier in the year?
  - How have your thoughts, feelings, and behaviour varied from those during the 1^st^ lockdown period?
  - Can you describe any activities (behaviours) that you are engaging in now that you were not doing during lockdown?
  - What has been the wider impact on your family and friends?
  - Have you felt fearful about covid 19 (if so, has this fear increased or decreased since the first lockdown and if so what has influenced this change)
  - Have you felt vulnerable/at risk, is yes, can you explain why (and what actions you have taken to reduce your vulnerability/risk) If no can you explain why you don’t feel vulnerable .
  - What measures are you using to in lockdown to help keep yourself safe (e,g. mask wearing), are you still using these measures and how do you feel about using them?
  - The term clinically extremely vulnerable has been used to describe people at high risk-what are your thoughts about this?
  - What effect has the media’s reporting of the pandemic had on your behaviour?
- The next set of questions are about any consultations you have had for your arthritis or any other medical conditions
- If you have attended for an appointment have you noticed any measures that have been taken to try to reassure you and make you feel safe (if so what have these been?)
- How effective do you feel the measures you have noticed have been at keeping you safe?
- Since our last interview have you had a consultation over the telephone or via a computer screen/video about your arthritis or other health conditions?
  - what worked well, what are the benefits to you?
  - what didn’t work so well, for example some people like to have someone present at a consultation (e.g. partner, family, friend), is this something that is important to you (if so, why)
  - how did/do feel about having the consultation on your own?
  - If you have had or were going to have a telephone or video consultation would it be important to you that it was with a doctor or health professional that you know (If yes why, if no why)
- What kind of information have you been accessing about your arthritis and what do you think about it
- Is the internet something that you (or a family member) have used to access information about your arthritis?
  - Has your use of the internet changed as a result of the pandemic?
  - Have you had to develop new IT skills to access information, if so what has this involved?
  - How challenging have these new skills been to develop
  - Has there been any help from family members/friends to develop new skills
  - Have you had to develop new skills in other areas during the pandemic (e.g booking appointments on line?, accessing on line exercise classes, video meetings with friends)
- The UK Government has approved a vaccination programme throughout the UK
  - Do you plan to have the vaccine? (explore response)
  - Will having the vaccine change you behaviour?, if yes, how
  - Should people with RA be a priority for a vaccine?
  - Do you think the vaccine will be effective for you?
- Is there anything that we have not discussed that you would like to share about your experience?
