# Supplementary material for: Perceptions of risk in people with inflammatory arthritis during the COVID-19 pandemic
Source: Rheumatol Adv Pract. 2022 Jun 20;6(2):rkac050. doi: 10.1093/rap/rkac050 (PMC9255274; doi:10.1093/rap/rkac050)
Supplement: rkac050_Supplementary_Data [file rkac050_supplementary_data.zip › 22-028 Supplementary Data S3 - 3rd stage interview guide.docx]

**Topic Guide**

**The experience of living with Inflammatory Arthritis during the Coronavirus Pandemic**

**Preamble**

- Since our last interview on (date of previous interview):
  - Can you describe any current impact on your physical, emotional, or social wellbeing due to your arthritis?
  - How has your arthritis been since our last interview, has there been any changes in your symptoms, and if so how have you managed those changes?
- Some people we have talked to are working (e.g. in employment, or dong voluntary work), if that applies to you, can you tell me your experiences since the last interview?
- Overall, how would you describe the impact that Covid has had on your RA
  - What has been the biggest impact on you as a result of Covid?
- We have had several periods of national and local restrictions, including a lengthy national restriction that started in December that has just started to relax.
  - How have your thoughts, feelings, and behaviour varied over time in terms of your management of risk of Covid?
  - If you have felt fearful at any time during the pandemic, how has that feeling altered now we are coming out of lockdown?
  - Have you felt vulnerable/at risk, is yes, can you explain why (and what actions you have taken to reduce your vulnerability/risk)?
  - Some people are no longer classified as having the need to shield, if this applies to you how does that make you feel, has this changed your behaviour?
  - How do you feel that potentially society is now in the process of re-opening, will this affect your own assessment of risk and vulnerability?
  - What are your thoughts about the future?
- The next set of questions are about any healthcare engagement you have had for your arthritis or any other medical conditions
- What is your assessment of healthcare (for your RA or any other health condition) over this period of Covid 19?
- If you could speak to the chief executive or lead of your health authority, what might you say?
- Are there things that healthcare could have done better during the pandemic, what might that be?
- These questions are about the use of technology (telephone or via a computer screen/video) for remote consultations about your arthritis or other health conditions?
  - Have you been involved in remote consultations related to your health, if so what was the experience like for you?
    - If so, what worked well, what are the benefits to you?
    - what didn’t work so well
  - If you haven’t had a remote consultation, what would be your thoughts and feelings about this approach?
  - If you have had or were going to have a telephone or video consultation, what would be important for you?
  - Do you think that remote consultations can be a useful feature in the future care of your RA (explore response)?
- As discussed in the last interview the UK Government had approved a vaccination programme throughout the UK
  - Have you had your vaccine, and if so, have you had your full vaccination (i.e. two jabs)?
  - Has having the vaccine changed your behaviour? If not why not, if yes, how?
- This will be the final interview for this project and we are very grateful for your participation. We wanted to ask a few final questions.
  - Overall, what would you say if the greatest impact of Covid on you?
  - Has Covid led you to consider things differently, for example about you and your RA?
  - Do you think that Covid will have an impact on healthcare in the future? (explore response)
  - What has been your experience in taking part in this research project?
  - Are there any areas that you feel we should have covered but have missed?
