# Supplementary material for: Perceptions of risk in people with inflammatory arthritis during the COVID-19 pandemic
Source: Rheumatol Adv Pract. 2022 Jun 20;6(2):rkac050. doi: 10.1093/rap/rkac050 (PMC9255274; doi:10.1093/rap/rkac050)
Supplement: rkac050_Supplementary_Data [file rkac050_supplementary_data.zip › 22-028 Supplementary Data S4 - Data analysis.docx]

**Supplementary Data S4: Data Analysis**

The following steps were undertaken to analyse the data as advocated by Braun and Clarke (2006).

1. Familiarisation with the data which involved repeated reading of the transcripts and noting of initial codes.
2. Generation of initial codes: coding relevant experiences from the data.
3. Identifying themes: by examining the coded data to identify specific patterns of meaning.
4. Review of themes: checking that the themes represent the data.
5. Defining and naming themes.
