# Supplementary material for: Perceptions of risk in people with inflammatory arthritis during the COVID-19 pandemic
Source: Rheumatol Adv Pract. 2022 Jun 20;6(2):rkac050. doi: 10.1093/rap/rkac050 (PMC9255274; doi:10.1093/rap/rkac050)
Supplement: rkac050_Supplementary_Data [file rkac050_supplementary_data.zip › 22-028 Supplementary Data S1 - 1st stage interview guide.docx]

**Topic Guide**

**The experience of living with Inflammatory Arthritis during the Coronavirus Pandemic**

**Introduction**

Discuss consent procedure (remind participant of study, consent given and right to withdraw), invite any questions about study, confirm verbal consent to take part.

Explain - No right or wrong answers within this interview, this is about your experiences and your thoughts which will be hugely valuable to our project.

Explain - The interview should roughly be about an hour in total. If at any time you wish to take a break (e.g. make a cuppa) then that is fine, I can stop and restart no problem.

Explain – my background is in research and I have no expertise within rheumatology and so would not be able to offer any clinical advice

**Participant profile**

- How long have been attending rheumatological services
- Occupational status
- Living situation (living alone/with family/other)

**Topic guide**

- When you first were aware of COVID did you take any extra precautions
  - When did you become aware?
  - At that time what did you understand to be a risk, where did you get your information on risk?
  - Was the information about risk clear for you to understand, were you confident about risk and precautions?
- Can you describe your experience of living with arthritis during this period (i.e. the period of lockdown)?
  - consider physical, general health
  - psychological (anxiety, depression)
  - social (impact of social participation, impact on social interaction with family, friends, groups)
  - occupational impact (ability to work, understanding from employers, financial impacts)
  - describe what a typical day involved during lockdown and what differences are there now
- Can you think of any specific impact there has been on your arthritis?
  - Treatment and ability to engage with rheumatology
  - Coping with your arthritis (physically, mentally)
- Can you share what it has been like to try and manage your arthritis during this time?
  - has the way you manage your arthritis changed if so how
  - what has been the key challenges and the key benefits (if any)
- Have you sought help for your arthritis, if so can you tell me about this?
  - use of telephone/internet sources of support (is this new for you or have you used before)
  - What have been the benefits and challenges of using remote contact
  - Could things have been done differently or better?

- Can you describe whether your health needs have changed?
- Can you describe any contact you have had with the rheumatology service during the period of lockdown and since?
  - consider remote consultations
  - adviceline (telephone and emails)
  - community blood tests
  - explore their perceptions of these, what was helpful/unhelpful, were they frequent enough, what was the quality like, could they have been better
- Can you think of any support that would have been helpful for you to receive from the rheumatology team during this period?
  - What could have worked better
- Have there been any advantages/unexpected benefits as a result of Covid-19
  - For you and your arthritis
  - With the engagement with rheumatology
  - For your general health and wellbeing
- What are your thoughts about the future?
  - Do you think that Covid-19 will have a long lasting impact on your arthritis
  - Do you think that you are better prepared now for something similar in the future?
- Is there anything else you would like to share about your experience?
  - For example the impact lockdown and shielding may have led to other people helping out more (friends, family, agencies), how did you feel about that?
- Is there anything that you thought I would ask about but haven’t?

The project plans to conduct follow up interviews in about 3 months, are you OK that we will contact you again to see how things are?
